# Deep learning techniques for Alzheimer's disease detection in 3D imaging: A systematic review

**DOI:** 10.1002/hsr2.70025

**Published:** 2024-09-18

**Authors:** Zia‐ur‐Rehman, Mohd Khalid Awang, Ghulam Ali, Muhammad Faheem

**Affiliations:** ^1^ Faculty of Informatics and Computing Universiti Sultan Zainal Abidin (UniSZA) Terengganu Malaysia; ^2^ Department of Computer Science University of Okara Okara Pakistan; ^3^ School of Technology and Innovations University of Vaasa Vaasa Finland

**Keywords:** 3D imaging, Alzheimer's disease, deep learning, supervised learning, machine learning

## Abstract

**Background and Aims:**

Alzheimer's disease (AD) is a degenerative neurological condition that worsens over time and leads to deterioration in cognitive abilities, reduced memory, and, eventually, a decrease in overall functioning. Timely and correct identification of Alzheimer's is essential for effective treatment. The systematic study specifically examines the application of deep learning (DL) algorithms in identifying AD using three‐dimensional (3D) imaging methods. The main goal is to evaluate these methods' current state, efficiency, and potential enhancements, offering valuable insights into how DL could improve AD's rapid and precise diagnosis.

**Methods:**

We searched different online repositories, such as IEEE Xplore, Elsevier, MDPI, PubMed Central, Science Direct, ACM, Springer, and others, to thoroughly summarize current research on DL methods to diagnose AD by analyzing 3D imaging data published between 2020 and 2024. We use PRISMA (Preferred Reporting Items for Systematic Reviews and Meta‐Analyses) guidelines to ensure the organization and understandability of the information collection process. We thoroughly analyzed the literature to determine the primary techniques used in these investigations and their findings.

**Results and Conclusion:**

The ability of convolutional neural networks (CNNs) and their variations, including 3D CNNs and recurrent neural networks, to detect both temporal and spatial characteristics in volumetric data has led to their widespread use. Methods such as transfer learning, combining multimodal data, and using attention procedures have improved models' precision and reliability. We selected 87 articles for evaluation. Out of these, 31 papers included various concepts, explanations, and elucidations of models and theories, while the other 56 papers primarily concentrated on issues related to practical implementation. This article introduces popular imaging types, 3D imaging for Alzheimer's detection, discusses the benefits and restrictions of the DL‐based approach to AD assessment, and gives a view toward future developments resulting from critical evaluation.

## 1 INTRODUCTION

**Figure 1:**
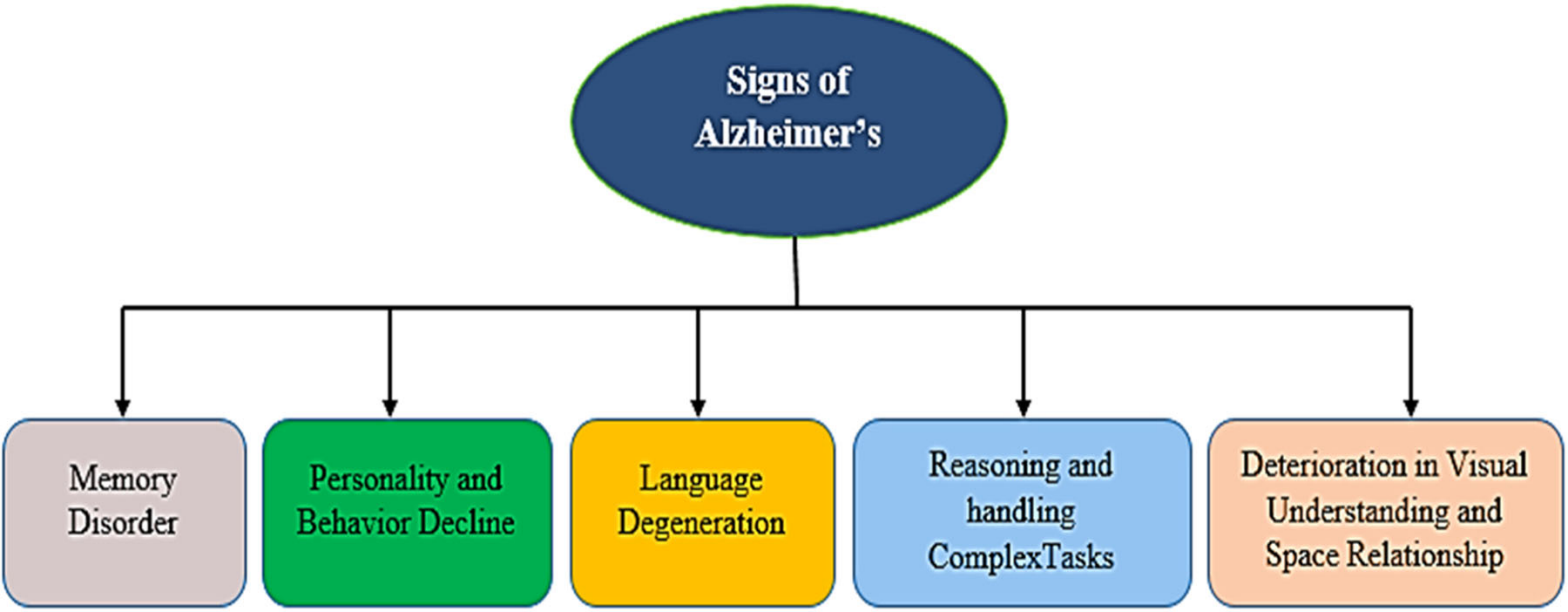
Different sings of Alzheimer's.

Dementia is a collection of diseases with increasing signs and symptoms, with Alzheimer's disease (AD) being the most common. AD is a medical condition defined by a gradual decline in thinking and memory functions. It is a prevalent condition amongst older individuals, constituting around 60%–80% of dementia cases. The recurrence rate of AD is substantial; however, no remedy is available. Over five million individuals globally have dementia, with 70% being AD people. The global frequency of AD was 30 million in 2006 but is expected to triple by 2050.^1^ The intensity of AD could lead to the death of the person. During the early phase, memory impairments are relatively minor.^1^ Figure 1 shows the different signs/indications of AD. However, as AD progresses, victims gradually lose their capacity to communicate and interact with their environment. In the later phases of the disease, this can ultimately result in a complete loss of senses for a long time.

**Figure 2:**
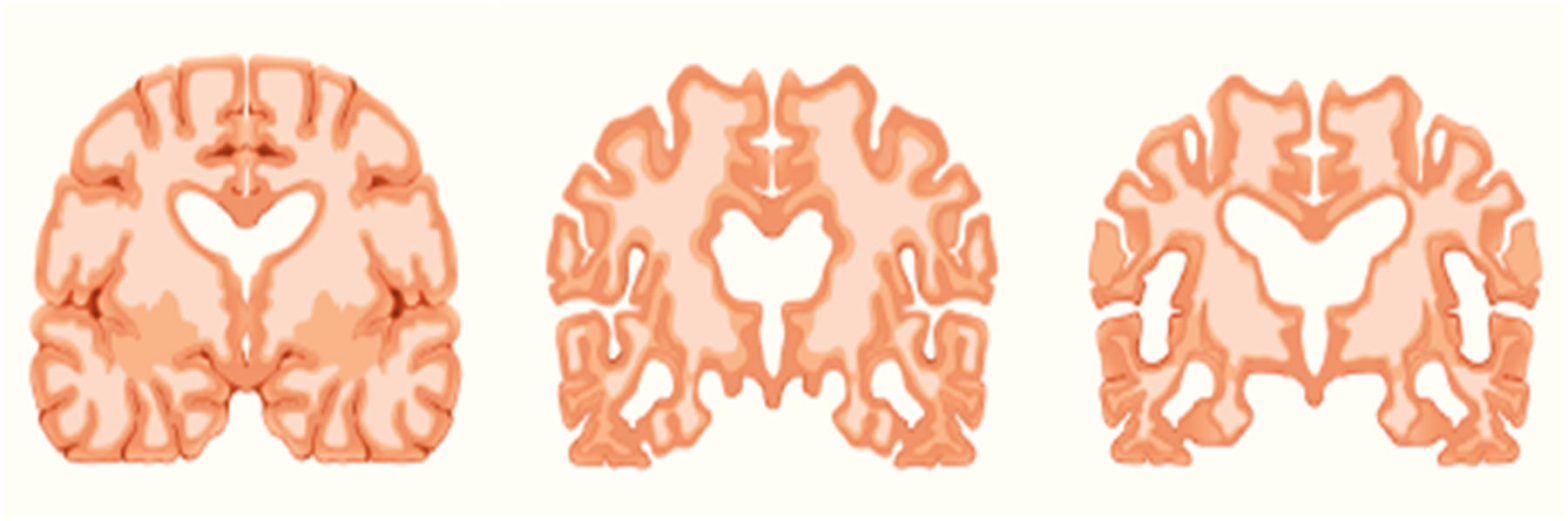
Alzheimer's stages from left to right are normal, mild, and severe.^3^

Initial detection of the disease facilitates appropriate patient treatment, therefore reducing the detrimental impact of AD and slowing the progression towards dementia.^2^ Figure 2 shows the different stages of AD.

**Figure 3:**
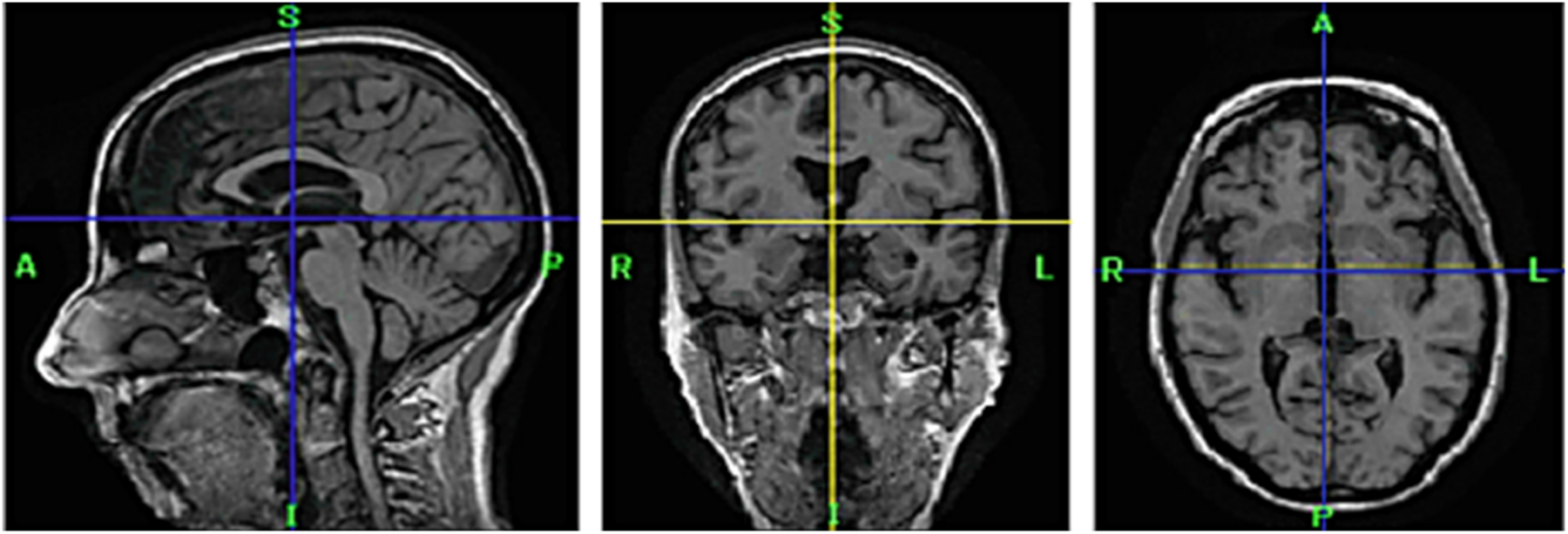
A three‐dimensional brain magnetic resonance imaging shows three planes (sagittal, coronal, and axial) from left to right.^13^

Magnetic resonance imaging (MRI) can differentiate between gray matter (GM) and white matter (WM). The hippocampus is a vital brain area for memory and learning.^4^ The hippocampus shrinks dramatically during mild cognitive impairment (MCI) to AD.^5^ MRI is the most commonly utilized method for neuroimaging due to its lack of radioactive tracer substances or hazardous gamma rays, distinguishing it from other forms of imaging.^6^ Treatment could stop the development of disease symptoms. Therefore, researchers in this field apply deep learning (DL) approaches.^7^, ^8^ DL is data‐hungry; as more data is added, DL algorithms become more effective and surpass conventional approaches like the human brain.^9^, ^10^ Earlier studies have shown a leading use of two‐dimensional (2D) images with DL techniques. Due to spatial information loss in the brain's cube, 2D MRI scan slicing has drawbacks.^11^ Previously, using 2D data, which converts three‐dimensional (3D) MRI images into 2D slices and uses them for feature extraction, was typical. Converting 3D to 2D loses feature detail. Straight 3D feature extraction may reveal information about the features because 3D input improves system efficiency.^12^ The three planes of 3D neuroimaging MRI are shown in Figure 3.

The majority of previous research on AD detection used 2D images, with very little research using 3D images.^14^ The latest developments in neural network topologies, data enhancement methods, and powerful GPUs allow 3D deep learning to interpret voxel clinical data. Therefore, throughout the past decade, there has been a significant increase in the use of 3D deep learning in various medical imaging techniques. In this study, we provide a comprehensive analysis of the uses of DL approaches with 3D imaging for diagnosing AD and potential areas for further research. As far as we know, research articles are available online. However, this is the first review study explicitly focusing on DL techniques used with 3D imaging for AD detection.

### 1.1 Search strategy

**Figure 4:**
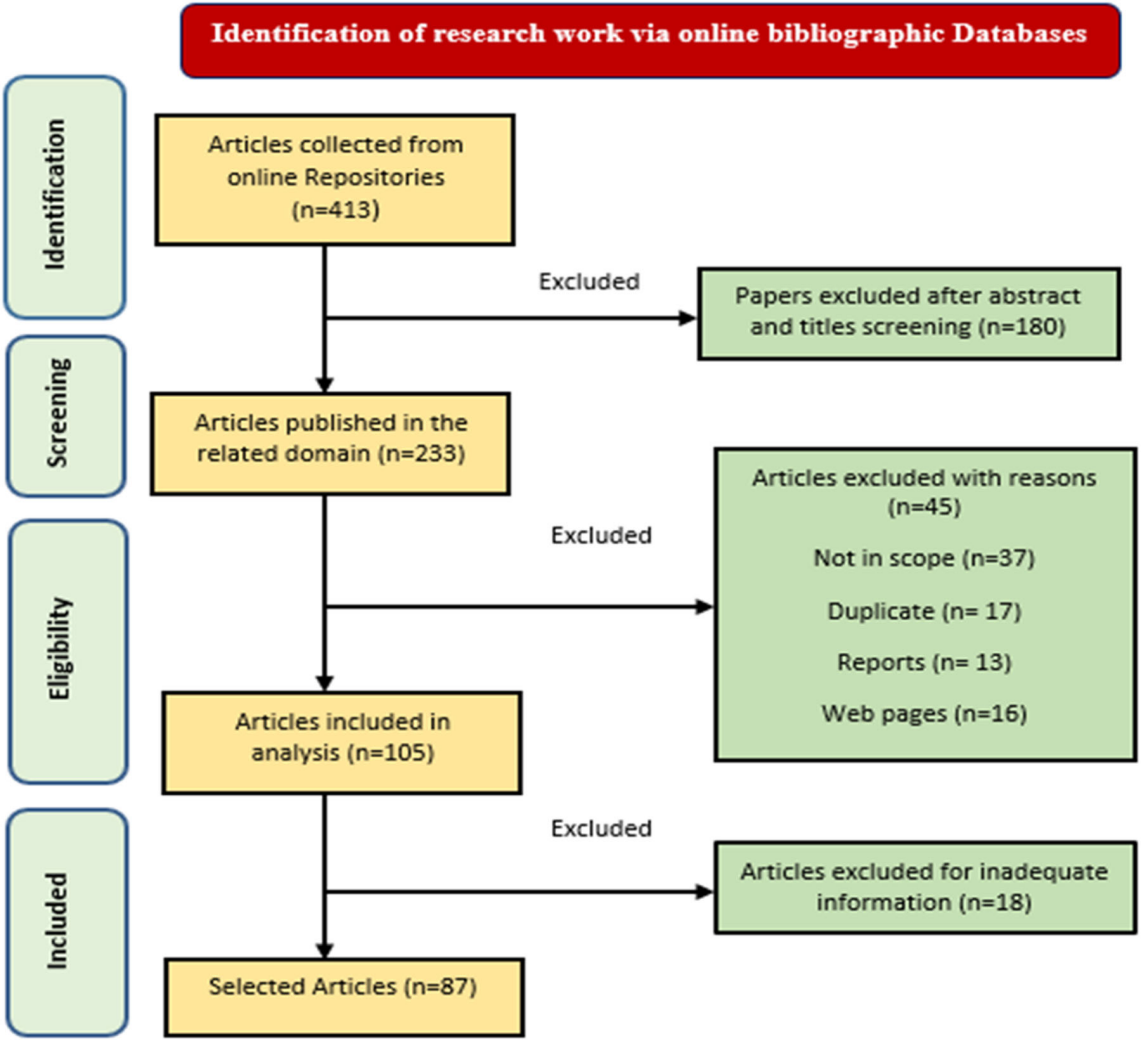
PRISMA flow diagram of the article selection procedure.

The research examines the acknowledgment of efforts to detect AD in several scientific databases, including IEEE Xplore, Elsevier, MDPI, PubMed Central, Science Direct, ACM, Springer, and other electronic library databases. The analysis focuses on papers that include keywords such as “deep learning in Alzheimer's,” “Alzheimer's diagnosis using deep learning,” “Alzheimer's detection using deep learning with 3D imaging,” and “Alzheimer's diagnosis and prediction using supervised deep learning.” We searched for research articles regarding AD detection using DL, which can be up to 25 pages in Google Scholar. Using the keywords mentioned above and reputable journals, we queried the Google Scholar database to include all publications, including those not found in the specified repositories. In this review article, we examine articles from 2020 to 2024. This research method follows the rules in Figure 4 of the PRISMA guidelines, ensuring the information collection process is organized and understandable. Moreover, Table 1 provides comprehensive information about the criteria for including and excluding data in PRISMA. This table thoroughly summarizes the criteria for deciding whether to review or exclude an item from the examination. At the onset of the study, we gathered papers from online database searches and chose 413 articles for the review.

**Table 1:** The table describes the inclusion and exclusion criteria for selected research papers.

	Inclusion criteria	Exclusion criteria
Research nature	Original research articles	Thesis, communication letters, white papers, editorials, and reports
Language applied	Research papers are presented in English.	Duplicate and non‐English research articles
Publication years	Articles published from 2020 to 2024 (for applications and results analysis part)	Not related to the subject of the review
Intervention	DL and 3D images	Traditional and arithmetical methods
Source of articles	Articles published in academic journals and conferences.	Research papers that insufficiency of information.
Settings	DL in medical imaging.	Not in medical imaging settings.
Region	Not restricted to an exact region.	NA

Abbreviations: 3D, three‐dimensional; DL, deep learning; NA, not available.

Following a thorough analysis, we selected 87 publications for review. Among these, 31 articles presented different ideas, descriptions, and explanations of theories and models, while the remaining 56 articles mainly focused on application problems and current breakthroughs. Our dedication to delivering a sophisticated and innovative assessment has prompted us to focus on works published in recent years intentionally.

### 1.2 Previous research gaps

Previous research on the application of DL to find AD in 3D images mainly focused on using CNN models and other DL models to get features and do classification tasks. However, further research is necessary to explore the capabilities of more recent structures, primarily designed to handle 3D MRI images. It is also essential to do more research using heterogeneous data fusion approaches. These involve combining data from different imaging types, like structural magnetic resonance imaging (sMRI), functional MRI (fMRI), and positron emission tomography (PET) imaging. This integration aims to improve the reliability and precision of the algorithms used for detecting AD. Enhanced GPUs, suitable system hardware, and adequately designed algorithms can increase implementation reliability. The comprehensibility of DL techniques in this field requires further research, limiting their use in healthcare. Addressing these inconsistencies can enhance the efficiency of detection and treatment methods for AD.

### 1.3 Contributions

The review article has the following contributions:

▪ Implementation of DL methods with 3D imaging to detect AD from 2020 to 2024.
▪ 3D images help identify and characterize brain deterioration by providing a full view of the brain area.
▪ We used 3D imaging to improve spatial resolution and understand complicated structures from many perspectives, which aids in accurately detecting AD.
▪ We explored 3D imaging with DL techniques to identify AD and its stages, using different datasets, imaging modalities, and core findings.

This review further discusses popular neuroimaging modalities, 3D imaging with DL approaches for Alzheimer's detection, discussion, challenges in AD, conclusion, and future extensions in this area.

## 2 BRAIN IMAGING MODALITIES FOR ALZHEIMER'S

According to previous research, numerous imaging procedures exist to help identify AD. However, the most prevalent and well‐acknowledged methods among experts are PET and MRI. The text concisely explains how MRI works, including its many patterns and PET imaging. It also discusses how both techniques are used in AD classification.

### 2.1 MRI imaging

**Figure 5:**
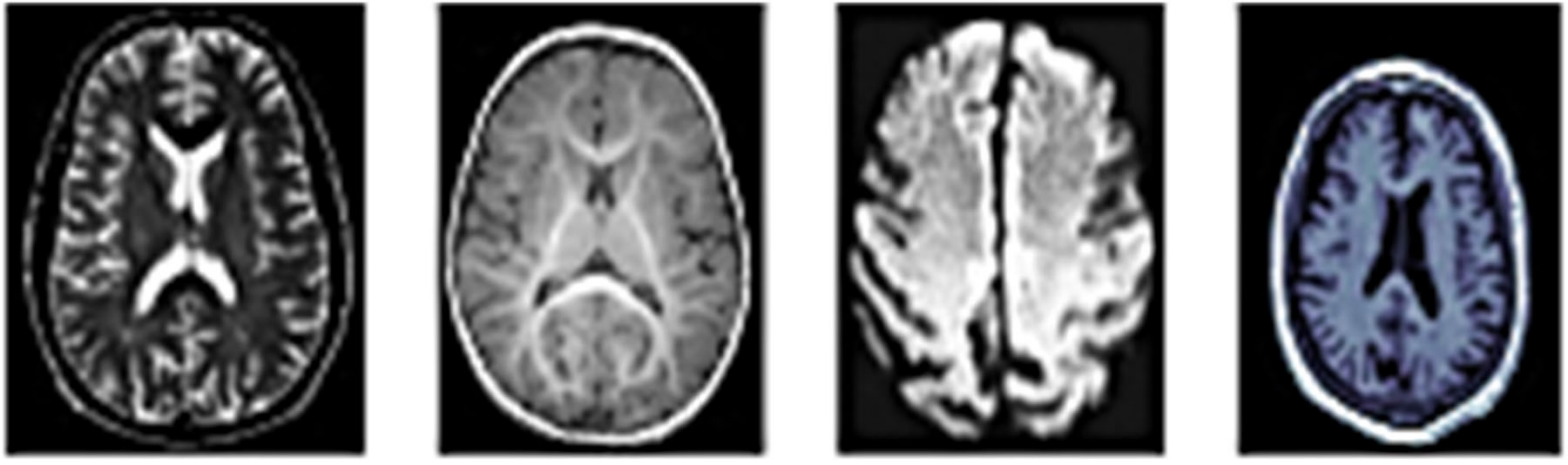
Different magnetic resonance imaging (MRI) sequence images were left to right: first (A) functional MRI (fMRI), (B) diffusion tensor imaging (DTI), (C) diffusion‐weighted imaging (DWI), and the last (D) T1‐W MRI.^15^

MRI is a significant advancement in clinical imaging. MRI provides excellent spatial detail and superficial tissue resolution, allowing for a 3D tomographic view and the ability to show how the body changes constantly.^6^ Figure 5 presents a variety of MRI series images.

The application of sMRI in the detection of AD is essential. This noninvasive imaging technique provides accurate information about the actual structure and condition of the brain as a whole.^16^ T1‐weighted MRI is utilized for structural evaluation and T2 for diagnostic investigation. Circulation variations in fMRI measure activity in the brain. Scientists often use fMRI to study neurological diseases such as bipolar disorder, and they are adopting it for further disease diagnosis.^17^ Diffusion tensor imaging (DTI) is an anisotropic diffusion technique employed in MRI to evaluate the organization of the brain's axonal tissue network. The last figure displays the maximum diffusivity of white‐matter fibers in this plane.^18^

### 2.2 PET imaging

**Figure 6:**
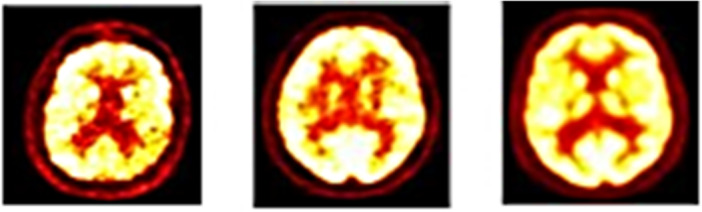
A positron emission tomography (PET) image of Alzheimer's disease (AD), cognitively normal (CN), and mild cognitive impairment (MCI) patients from left to right.

Researchers often utilize 18‐fluoro‐deoxyglucose (FDG) PET in addition to MRI to identify AD due to its capacity to improve training methods. Currently, PET imaging with molecules is available for use in healthcare. PET is commonly used for cell characterization, classification, therapeutic management, and cerebral glucose metabolism detection. PET frequently demonstrates the development of AD, including the phases of MCI.^19^ Figure 6 Alzheimer's stages on PET images.

Regular PET investigations are not feasible due to the radioactive elements employed in scanning. FDGPET is the most commonly used PET for detecting AD. The PET method requires immediate imaging after injecting the radioactive tracer substances, making it time‐sensitive. The device captures photons, creating 3D images of the subject.^20^ Electroencephalograms, MRIs, and PETs are standard neuroimaging techniques used to study brain function and make diagnoses. Since MRI and PET became unaffordable, EEG became the preferred diagnostic method.^21^

## 3 ALZHEIMER'S DIAGNOSIS WITH DL APPROACHES

DL's untapped potential is gaining significant interest in clinical studies. DL enables algorithms using computation and multi‐processing levels to acquire a collection of attributes from source data and classify the result based on the learned data.^22^ DL is suggested for initial AD detection due to its superior image accuracy for classification compared to the classic machine learning approach.^23^, ^24^ Unsupervised learning uses training data without labels. The following are the most commonly used unsupervised learning models:

▪ Autoencoder (AE)

▪ Deep Belief Network (DBN)
▪ Generative adversarial networks (GANs)
▪ Restricted Boltzmann Machines (RBMs)
▪ Variational Autoencoders (VAEs)

In supervised DL, methods are learned to map input information into output labels using pairs of inputs and outputs from the training process. The core models and their variants are primarily used as;

▪ Convolutional Neural Network (CNN)
▪ Recurrent Neural Network (RNN)
▪ Long short‐term memory (LSTM)
▪ Gated Recurrent Unit (GRU)
▪ Capsule Networks
▪ Transformers

**Figure 7:**
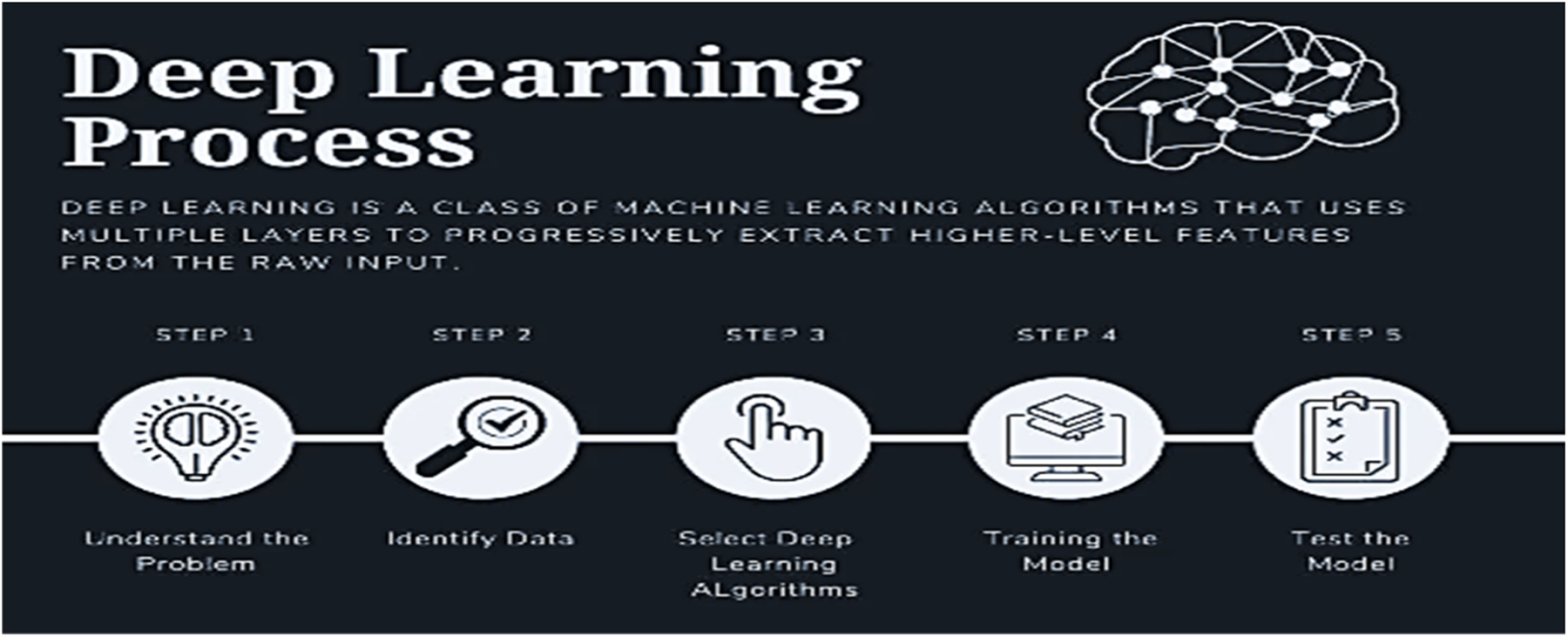
Different steps of deep learning algorithms.^25^

Here, we aim to define transformers as models that can be classified into supervised or unsupervised deep learning, depending on the specific application. Figure 7 shows the different steps of DL methods researchers used for their work.

The following DL with 3D image data for AD detection is described in detail.

### 3.1 3D imaging for AD detection

DL techniques use 3D images to evaluate and retrieve data for AD diagnosis. We present the details of various models used with 3D imaging in diverse domains. Table 2 presents the supervised DL with 3D imaging, which has the data set name, imaging modality, model, and critical findings.

**Table 2:** AD diagnosis research using supervised DL with 3D imaging.

Reference	Year	Data set	Image type	Method	Key findings
Begum and Selvaraj^26^	2023	ADNI	fMRI, MRI, DTI, and PET	DCNN	Feature investigation and classification
Parmar et al.^27^	2020	ADNI	fMRI	3D‐CNN	Extracted spatiotemporal features
Tuan et al.^28^	2022	ADNI and OASIS	MRI	CNN, XGBoost, SVM	Categorize AD based on the segmented tissues
Tufail et al.^29^	2022	ADNI	MRI, PET	3D‐CNN	Model engineering has a reduced effect on classification
Tomassini et al.^30^	2021	OASIS and ADNI	sMRI	Convolutional Long Short‐Term Memory	it can be used for other mental illnesses
Ayyar et al.^31^	2021	ADNI	sMRI	3D‐CNN	Hippocampal ROIs of the brain
Pardakhti and Sajedi^32^	2020	ADNI	MRI	3D‐CNN	Estimation of brain age
Chakraborty et al.^33^	2021	ADNI	MRI	3DCNN	Good performance measures
Folego et al.^34^	2020	ADNI	MRI	3D‐CNN	Automatic and relatively fast
Chen et al.^35^	2022	ADNI‐1 and ADNI‐2	MRI	Multiview‐slice Attention and 3D‐CNN	Slice‐level attention
Balboni et al.^36^	2022	Three hospital datasets	MRI	Transfer learning	Enhanced hippocampal segmentation feature
Rogeau et al.^37^	2024	ADNI, FTLDNI	18F‐FDG PET	3D VGG16	Excellent accuracy
İsmail and Dalveren^38^	2024	ADNI	fMRI	3D‐CAPSNET and RNN	Spatiotemporal information for AD
Lin et al.^39^	2024	ADNI	MRI	Pre‐trained ResNet	Enhances the method's training cost
Yang et al.^40^	2024	ADNI2	PET, MRI	Contrastive Masked Vim Autoencoder	The model improves by 2.7% AUC performance
Alp et al.^41^	2024	ADNI	MRI	Vision Transformer	Excellent classification accuracy
Zhang et al.^42^	2021	ADNI	MRI	Densely connected + attention mechanism	Excellent classification accuracy
Nasina and Reddy^43^	2020	ADNI	MRI	3D‐CNN	Good performance
Zhang et al.^44^	2021	ADNI	sMRI	3D Residual Self‐Attention DNN	Better Accuracy and generalizability
Dhinagar et al.^45^	2021	ADNI, OASIS	MRI	3D‐CNN	Generalized superior on unseen MRI
Nawaz et al.^46^	2020	ADNI	MRI	3D‐CNN	Good for imbalance data
Gao et al.^47^	2022	ADNI	sMRI	3D‐MgNe	Reduce the number of parameters
Narazani et al.^48^	2022	ADNI	PET, MRI	3D‐CNN	PET captures AD pathologies better
Zuo et al.^49^	2024	Huanhu Hospital, China	Eye‐tracking	Multilayered comparison CNN	Consistent validity in classifying AD patients and NC
Zhang et al.^50^	2023	ADNI	MRI, PET	Attention‐based 3D‐CNN	Emphasis on main brain regions linked to the disease.
Turrisi et al.^51^	2023	ADNI	MRI	3D‐CNN	Data augmentation approach plays an important part in performance
Ullanat et al.^52^	2021	ADNI	MRI	3D ResNet‐18	Effectiveness of models in identifying AD‐related brain areas.
Zheng et al.^53^	2023	ADNI	MRI	3D EfficientNet	High classification accuracy in identifying AD and detecting MCI progression.
Kong et al.^54^	2022	ADNI	PET, MRI	3D‐CNN	Get better multi‐modal feature
Agarwal et al.^55^	2022	ADNI	MRI	End‐to‐end‐learning deep CNN	Complicated MR image features that might enable early identification
Tufail et al.^29^	2022	ADNI	MRI, PET	3D‐CNN	Integrating augmentation approaches can decrease classification performance.
Etminani et al.^56^	2022	ADNI	PET	3D, DL	Every neurological disease needs a posterior cingulate cortex
Biswas and Gin^57^	2023	OASIS and ADNI	MRI	Random forest, KNN, Gradient boost, and decision tree.	Highest accuracy for WM volume using the OASIS data set
Wu et al.^58^	2022	OASIS	MRI	3D Transfer learning	The classification time is abridged.
Dyrba et al.^59^	2021	ADNI	MRI	3D‐CNN	Hippocampus shrinkage was the best AD indicator.
Kang et al.^60^	2023	ADNI	MRI	3D Deep Convolutional GANs	The model prevents overfitting due to insufficient sMRI data.
Zhang et al.^61^	2021	ADNI	MRI	3D‐CNN	Increases training speed and reduces boundary noise
Tufail et al.^62^	2022	ADNI	PET	CNN	Superior performance on the multiclass classification
Soliman et al.^63^	2020	ADNI	MRI	3D‐CNN	Develop general features for detecting AD biomarkers.
Kumari et al.^64^	2022	ADNI	MRI	3D‐CNN and modified owl search	Improving the weights of the suggested model
Nithya et al.^65^	2023	ADNI	MRI	3D‐CNN	Good prediction accuracy compared to other models
Akan et al.^66^	2024	ADNI	MRI	Vision Transformers and Bi‐LSTM	Good classification accuracy.
Tomassiini et al.^30^	2021	ADNI	sMRI	ConvLSTM	Offer a less data set‐specific method.
Chen et al.^67^	2024	ADNI, OASIS, and AIBL,	MRI	3D‐CNN, Longitudinal Transformer	Efficiently merge spatiotemporal features for classification.
Khan et al.^68^	2024	ADNI	MRI and PET	Dual‐3DM3‐AD method	Segmentation, efficiently decreasing complexity
Ningsen et al.^69^	2024	Kaggle	MRI	Multi‐Order 3D U‐NET, CNN	Considerably enhanced the segmentation accuracy.
Kim et al.^70^	2024	ADNI	MRI and PET	Multimodal 3D DL	Discovering the importance of ROI‐based techniques in refining effectiveness
Erdemir^71^	2024	ADNI	MRI	3D LSTM method	Little sensitivity and F1 score in detecting the shift from mild to AD
Uyguroğlu et al.^72^	2024	ADNI and AIBL	MRI	CNN and 3D angular orientations	Innovative usage of distinctive angles of the full data set by new perspectives
Ning et al.^73^	2024	ADNI	MRI and PET	3D‐CNN	Variations in cortical areas can detect
Castellano et al.^74^	2024	ADNI	MRI and PET	Multi‐modal and Uni‐modal methods	Concentrations on important Alzheimer's associated regions for its detection
Rahman et al.^75^	2024	OASIS‐3	CT	Transfer learning method	Extremely correct in identifying pelvic cracks
Dharwada et al.^76^	2024	ADNI	sMRI	3D Transfer learning	Weights in every ensemble method are tuned for enhanced accuracy
Li et al.^77^	2024	ADNI	MRI	New Med‐3D Transfer technique	Gives a dynamic platform for encountering real‐time problem‐solving
Saad and Hasan^78^	2024	ADNI	MRI	3D Vision Transformers	Understand the brain parts that greatly influence solving classification issues
Hu et al.^79^	2024	ADNI	MRI	3D‐SEConvNeXt model	Attaining excellent results in early AD diagnosis tasks

Abbreviations: CT, computed tomography; DTI, diffusion tensor imaging; fMRI, functional magnetic resonance imaging; MRI, magnetic resonance imaging; sMRI, structural magnetic resonance imaging; PET, positron emission tomography.

Different methods have been applied in the article, and each has its pros and cons regarding its structural nature. Table 3 shows the algorithms used in this article and their advantages and limitations.

**Table 3:** DL methods and their advantages and disadvantages.

Methods	Advantages	Disadvantages
3D‐CNN	Possibly will get 3D data from brain scans.	The training process is expensive in terms of different resources.
RNN	Suitable for continuous 2D photographs	The gradient vanishes and then strengthens rapidly.
AE	Capable communication abilities. It helps the decrease of dimensionality and is guileless to use.	Restrictions in adaptableness.
DBN	Deliver better‐ordered feature procurement.	Require significant computing resources for training procedures.
GANs	Produce correct distributions of data.	Tough to train and susceptible to method disintegration, which restricts generator efficiency.
DNN	Can control several datasets. Minimal feature engineering limits	The timing of training procedures is problematic, and weights are hard to comprehend.
Transformers	Competently control sequential data.	Challenging and expensive in training.
Caps Net	Improve the ordered learning of representations.	It is more computationally costly than CNNs.
RBM	Extremely expressive and favorable to rational reasoning.	The training process is resource‐intensive.

Abbreviations: AE, Autoencoder; 3D CNN, three‐dimensional convolutional neural network; DBN, Deep Belief Network; DL, deep learning; GANs, generative adversarial networks; RBM, Restricted Boltzmann Machines; RNN, recurrent neural networks.

### 3.2 Performance comparison of DL algorithms for Alzheimer's detection

When diagnosing Alzheimer's using 3D scans, different DL methods demonstrate a range of performance and advantages. CNNs are highly effective because they can identify spatial patterns in image data, making them widely preferred for clinical imaging tasks. Due to their specific structure for sequential data, we do not commonly employ LSTMs, RNNs, and GRUs for this objective. However, we can integrate them with CNN models to boost their effectiveness. GANs and VAEs are valuable methods for producing artificial 3D scans, improving data set training processes, and strengthening models' adaptability. Current research uses fewer RBMs and DBNs as more sophisticated designs emerge. Capsule networks, known for their ability to maintain spatial correlations, have potential but are currently in the early stages of research. Researchers are now investigating the possibility of transformers in 3D healthcare imaging, renowned for their ability to handle sequences and attention‐intensive tasks. This is mainly due to their impressive feature retrieval capability. CNNs, enhanced by GANs and VAEs, currently provide the highest level of performance in the AD field utilizing 3D imaging.

## 4 DISCUSSION

Current studies^15^, ^17^ show that DL approaches are becoming more helpful in identifying AD. These studies highlight the potential of DL algorithms, especially CNNs, in interpreting medical imaging data like MRI, PET, and CT images. Such algorithms can accurately distinguish AD from normal controls and the early stages of AD by automatically extracting and learning features from massive datasets. Researchers also combine images and clinical and genetic data to improve detection effectiveness. However, big, well‐annotated datasets, model interpretation, and comprehensive validation among varied populations are challenges. Despite these challenges, DL‐based approaches are becoming more popular for timely and precise AD diagnosis, ensuring enhanced patient outcomes with earlier management. This research highlights significant advancements in DL techniques to identify AD on time using 3D imaging modalities. DL algorithms have recently seen a substantial increase in AD diagnosis and prediction, demonstrating promising outcomes in many areas. 3D imaging improves visualization and perception of depth, facilitating healthcare diagnosis, architectural design, and simulations. Its comprehensive spatial data allows for better assessments and evaluations than 2D imaging. In future studies, better research, standardized imaging techniques, and model improvements' interpretability can help address these shortcomings.

## 5 CHALLENGES

Although DL techniques have yielded positive results, identifying AD still faces several issues that require attention. The data augmentation and transfer learning models have stopped overfitting problems in the research and group of information. However, issues with generalization can still happen when there are not enough data items.^80^ The data set will be expanded. However, its efficiency remains uncertain, and more studies will continue in this domain. Supervised DL has mitigated this issue and decreased the reliance on professional expertise, but additional research is necessary. Despite DL methods' impressive achievements, AD identification faces several restrictions and challenges.^81^ A comprehensive understanding of the depth method, benchmarking platform, and other relevant factors is necessary to determine the ideal balance of several biomarkers. Several fusion approaches have the potential to contribute to research on AD.^82^, ^83^ Still, there are obstacles to overcome when using DL‐based techniques^84^, ^85^, ^86^, ^87^, ^88^, ^89^, ^90^, ^91^ to tackle AD.

## 6 CONCLUSION AND FUTURE DIRECTIONS

The early identification of AD remains a challenge, and computer scientists are continuously investigating potential solutions. This work presents the biomarkers associated with AD, imaging modalities, and 3D imaging applications for AD identification. DL techniques have shown success in using multiple data modalities. Regarding classification algorithms, CNN is among those most often employed and outperforms other deep models in this area. Even though most issues in AD classification remain unsolved, the problem of overfitting the data set requires solutions. Given the shortage of healthcare data, unsupervised and self‐monitoring approaches are becoming prominent areas of study in medical imaging. To advance precision treatment in AD management, robust DL algorithms that can extrapolate within diverse populations use multifaceted data integration for disease identification and easily integrate into clinical operations through transparent and comprehensible decision‐support platforms.

## AUTHOR CONTRIBUTIONS

**Zia‐ur‐Rehman**: Writing—original draft; software; data curation; resources; conceptualization. **Mohd Khalid Awang**: Formal analysis; validation; investigation; supervision. **Ghulam Ali**: Conceptualization; writing—review and editing; methodology; project administration. **Muhammad Faheem**: Methodology; data curation, formal analysis; visualization, review and editing.

## CONFLICT OF INTEREST STATEMENT

The authors declare no conflict of interest.

## ETHICS STATEMENT

We used the Preferred Reporting Items for Systematic Reviews and Meta‐Analyses (PRISMA) guidelines for our study. This study did not include any actual human or animal subjects. We assess all studies that undergo review according to ethical principles.

## TRANSPARENCY STATEMENT

The lead author Muhammad Faheem affirms that this manuscript is an honest, accurate, and transparent account of the study being reported; that no important aspects of the study have been omitted; and that any discrepancies from the study as planned (and, if relevant, registered) have been explained.

## Data Availability

Data sharing is not applicable to this article as no datasets were generated or analyzed during the current study. Data that was analyzed is available from the corresponding author upon reasonable request.
